# Supplementary material for: Splice-Junction-Based Mapping of Alternative Isoforms in the Human Proteome
Source: Cell Rep. Author manuscript; Available in PMC 2020 Jan 15. (PMC6961840; doi:10.1016/j.celrep.2019.11.026)
Supplement: 3 [file NIHMS1546469-supplement-3.zip › DF2/PXD009737/TestisGluC-72-Q13813-QGSIALRQEQIDNQYHSLLE.pdf]

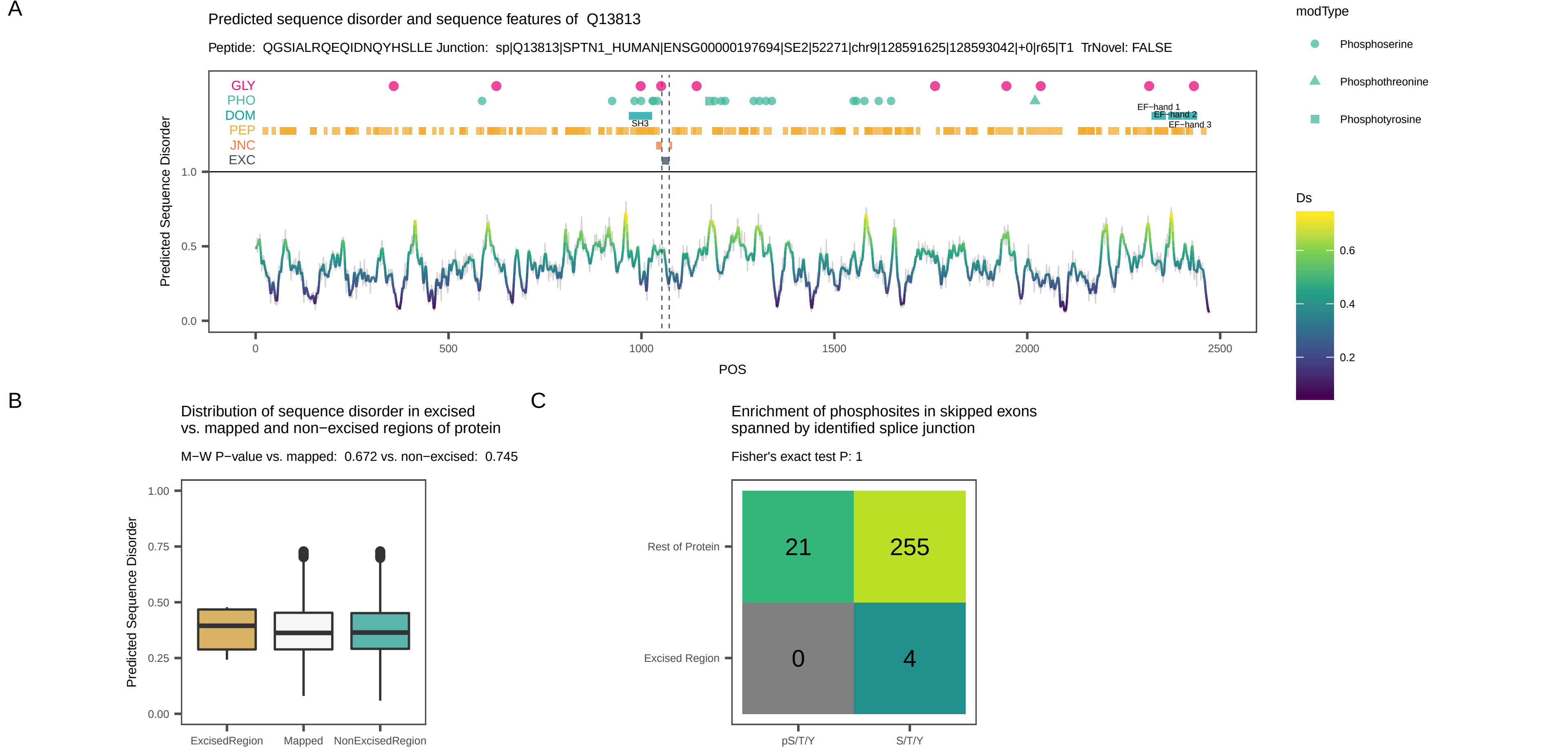

A
Predicted sequence disorder and sequence features of Q13813
Peptide: QGSIALRQEQIDNQYHSLLE Junction: sp|Q13813|SPTN1_HUMAN|ENSG00000197694|SE2|52271|chr9|128591625|128593042|+0|r65|T1 TrNovel: FALSE
GLY
PHO
DOM
PEP
JNC
EXC
SH3
EF−hand 1
EF−hand 2
EF−hand 3
Predicted Sequence Disorder
POS
modType
Phosphoserine
Phosphothreonine
Phosphotyrosine
Ds
B
Distribution of sequence disorder in excised vs. mapped and non−excised regions of protein
M−W P−value vs. mapped: 0.672 vs. non−excised: 0.745
Predicted Sequence Disorder
ExcisedRegion
Mapped
NonExcisedRegion
C
Enrichment of phosphosites in skipped exons spanned by identified splice junction
Fisher's exact test P: 1
Rest of Protein
Excised Region
21
255
0
4
pS/T/Y
S/T/Y
